# Comparison of Boer, Kiko, and Spanish meat goat does for stayability and cumulative reproductive output in the humid subtropical southeastern United States

**DOI:** 10.1186/1746-6148-8-136

**Published:** 2012-08-17

**Authors:** Ashley N Pellerin, Richard Browning

**Affiliations:** 1 Institute of Agricultural and Environmental Research, Tennessee State University, Nashville, USA

**Keywords:** Meat goats, Boer, Longevity, Lifetime performance, Adult, Mortality, Reproduction

## Abstract

**Background:**

Longevity is the amount of time breeding females stay active in a herd by avoiding death or culling because of illness or reproductive failure. This is a trait of economic relevance in commercial small ruminant breeding herds as it affects lifetime reproductive output. The purpose of this study was to determine if breed of meat goat influences breeding doe survival rates and cumulative reproductive performance under semi-intensive management.

**Results:**

Boer (n = 132), Kiko (n = 92) and Spanish (n = 79) does were evaluated for longevity trends and cumulative kid production. The herd was managed on humid subtropical pasture. Does had the chance to complete 2 to 6 production years. Survival curves were analyzed for 2 culling methods. The actual culling practice removed does after two failures to wean a kid. An alternative culling protocol removed doe records after the first failure to wean a kid. Kid production traits analyzed across herd life were the total number of kids weaned and cumulative kid weight weaned to the 2-, 3-, and 5-year stayability endpoints. Most (82%) doe exits were illness-related under the actual culling method. Reproductive failure represented 51% of doe exits under the alternative culling protocol. Boer does had greater survival declines (*P* < 0.01) from 2 to 6 years of herd life compared with Kiko and Spanish under both culling protocols. Boer does had lower stayability rates (*P* < 0.01) at each year endpoint for both culling protocols. Under the alternative protocol, over 50% of Boer does failed to complete 2 years, whereas over 50% of Kiko and Spanish does successfully completed 4 years. Boer does had lower (*P* < 0.01) total number of kids weaned and cumulative weight weaned through each stayability endpoint compared with Kiko and Spanish.

**Conclusion:**

Boer does had low stayability and cumulative kid production rates compared with Kiko and Spanish does. Poor health was the primary driver of does exiting the herd. Kiko and Spanish does did not differ for longevity and lifetime performance indicators.

## Background

Stayability is an indicator of longevity that measures the proportion of animals remaining productive to a fixed time endpoint [1]. Functional longevity (i.e., length of productive life) is defined by the ability of animals to avoid death or culling as a result of illness or reproductive failure. Longevity of breeding females has an economic impact on commercial meat animal production systems [2-4]. Females with short productive lives are costly in terms of lost production at peak performance ages, biological and economical costs associated with young replacements, medical treatments associated with subsequent illness culls, and lost salvage value in cases of death. Females with extended productive lives would be expected to generate a greater lifetime amount of marketable product (e.g., offspring, milk, etc.) with associated herd-wide benefits. Published studies of longevity in goats are few and limited to dairy herds [5-7]. In the US, maintaining adequate health in meat goats is a greater challenge than in other livestock classes because of the relative lack of medications approved for use in goats by the US Food and Drug Administration (FDA). For example, internal parasitism is widely recognized as a primary health risk to sustainable goat production [8]. However, only four anthelmintics are FDA-approved for goats (three bendazole products and morantel tartrate). Widespread endoparasite resistance to bendazoles is evident in goat herds [9,10]. Morantel tartrate is available as a feed additive; however, feed is not viewed as a preferred delivery method for anthelmintics in goat herds [11].

Genetics is often overlooked aspect of herd health management. Breed selection and utilization are important in enterprise success, particularly in the area of doe performance. Evaluation of traits that indicate female fitness is often not considered when comparing meat goat breed options. There are 3 primary meat goat breeds managed in the US. The Boer goat is native to South Africa [12]. It has become the most popular meat goat in the US since its introduction in the 1990s. The Spanish goat is a landrace genotype that has evolved from centuries of natural selection in the US [13]. Spanish goats were the predominant type until the Boer was imported. The Kiko goat was developed in New Zealand and introduced to the US during the same time period as the Boer. The composite Kiko breed resulted from a series of crosses involving feral does and varying breeds of dairy bucks [14]. Differences between breedtypes for longevity and cumulative productivity are reported in ovine ewes [15-17]. Similar breed comparisons have not been reported for meat goat does. Boer does had lower annual reproductive rates and higher annual attrition rates compared with Kiko and Spanish does in a low- to moderate-input, pasture-based production system [18]. Research had not been conducted to determine how the breeds may rank for annual performance in an intensive, high-input production system or if any relevant genotype x environmental interactions exist. Reported here are the comparative values of Boer, Kiko, and Spanish does for longevity and lifetime kid production traits when semi-intensively managed on pasture.

## Methods

### Study location

The herd was maintained at the Tennessee State University research station along the Cumberland River in Nashville, Tennessee (36.176°N, 86.828°W). The station is in the humid, subtropical southeastern United States and has an annual precipitation total of 1,222 mm evenly distributed throughout the year. The mean annual temperature is 15°C. Seasonal temperature extremes are represented by average summer daytime highs of 32°C in July and winter nighttime lows averaging −2°C in January. The 12-mo precipitation total during the project ranged from a high of 1,434 mm in 2004 to a low of 790 mm in 2007.

### Study animals

Straightbred does represented Boer (n = 132), Kiko (n = 92), and Spanish (n = 79) meat goat breeds. The study does were the daughters of 86 Boer sires, 28 Kiko sires, and 17 Spanish sires. Table 1 presents the incoming does for each entry year. The production year extended from September (start of fall breeding) to August (end of summer weaning), thus the production year is represented by the calendar year of kidding in this report. The foundation herd consisted of purchased does that entered the breeding program in 2004 and 2005. Herd entries in 2004 included some Boer and Kiko does [19] along with Spanish does that were part of program establishment the previous production year. Data from 2003 establishment year were not considered in the current dataset and those does were treated as new herd entries in 2004 for this dataset. Entries in 2006, 2007, and 2008 were replacement does produced within the research herd except for a few purchased Boer replacement does in each year. Purchased does were generally 2 to 3 years of age at first kidding season in the project herd for all 5 years of entries (Table 1). Some purchased does within each breed had parturition opportunities before entering the study herd, including those from the earlier pilot study [19]. All replacement does produced within the research herd were the daughters of the 22 Boer service sires, 18 Kiko service sires, and 14 Spanish service sires used over the six-year study. Random doe breeding assignments resulting in sire-daughter, dam-son, or full sibling matings were nullified and a new doe breeding assignment was generated. This was done in an effort to minimize inbreeding. The replacement does born and raised on the research station were 2 years of age at first kidding season. Within-breed diversity of genetics and herd sources was broad to provide a representative sampling for each breed [18].

**Table 1:** Number and average age of new does entering the herd each year per breed

	**Boer**		**Kiko**		**Spanish**	
**Entry year**	**does, n**	**age, yr**	**does, n**	**age, yr**	**does, n**	**age, yr**
2003-04	41	2.2	38	2.2	47	2.6
2004-05	26	2.4^a^	13	2.2	4	2.0
2005-06	14	2.0	13	2.0	8	2.0
2006-07	34	2.1	11	2.0	11	2.0
2007-08	17	2.0	17	2.0	9	2.0

^a^The maximum age of doe entries for any group was 3 years except for the 2004–05 Boer entry group in which four 4-yr-old does were added to the herd.

### Herd management

Does grazed cool season tall fescue (*Festuca arundinacea)* and warm-season bermudagrass (*Cynodon dactylon)* pastures. A variety of other grasses, clovers, broadleaf weeds, and woody browse species were available in grazing areas. Supplemental orchardgrass hay (*Dactylis glomerata)* was provided for *ad libitum* consumption throughout the year along with free access to water and mineral supplement. Does were provided with a 16% crude protein pelleted supplement periodically throughout the study; primarily 454 g/d during breeding and lactation. Stocking densities were approximately 12 does per hectare.

Does were bred to kid once a year in a traditional fall-breeding, spring-kidding scenario using a full 3-breed diallel-mating scheme. Breeding was by single-sire natural mating with 4 to 6 weeks of buck exposure. Bucks were selected based on the potential to provide genetic and source diversity to the program. Performance information was not used in the acquisition of purchased bucks. Bucks produced and developed within the research herd were those that ranked high for weaning weight ratios. Bucks stayed in the herd sire battery for one to five years. Bucks were removed for health reasons or were replaced by new bucks that introduced new genetic line for increased genetic diversity. The herd was split into a March-kidding management group and a May-kidding group each year. Does kidded on pasture with access to shelter. Kids were not creep-fed and male kids were not castrated. Kids suckled dams until weaned at a contemporary group median age of approximately 90 days. At weaning, kids were dewormed and vaccinated against *Clostridium perfringens* Types C and D, tetanus, and pneumonia. All straightbred doelings were retained and developed as herd replacements.

Does were vaccinated against *Clostridium perfringens* Types C and D and tetanus during the fourth month of gestation and dewormed at parturition in each year. Does were also dewormed before or after fall breeding in the first 3 years. In the last 3 years of the study, the pneumonia vaccine was administered to does at 4 months of pregnancy. All other health treatments were provided to individual does based on observation of clinical symptoms of disease. Daily checks were made to evaluate herd health status. The primary health issues recorded and treated were lameness and internal parasitism. Other doe herd health issues occurred infrequently, including pneumonia and dystocia. The culling protocol during the study removed does from the herd after the second failure to wean a kid or of chronic illness. Annual reproductive records were maintained and reviewed to determine when a doe failed to wean a kid the first time and when she had a subsequent year of weaning failure. Does also left the herd due to deaths resulting from illness, reproductive complications or accidents. Records were maintained on all health treatments, culls, and deaths. Additional details of doe and kid management are presented in previous reports [18,20]. Herd management protocols used on this study were approved by the Tennessee State University Animal Care and Use Committee as conforming to established standards for agricultural animal management in research.

### Statistical procedures

Stayability was measured to 2, 3, and 5 years of possible herd life (i.e., years of doe production). Unless otherwise indicated, references to ‘years’ in presenting stayability results of the current study generally refer to years of possible herd life and not doe age or project years. Does remaining in the herd at the conclusion of the study in August 2009 were considered censored and coded as 0 and does that exited the herd were coded as 1 for each stayability endpoint. An alternative culling protocol was also evaluated by removing doe records from the active herd dataset after the first failure to wean a kid. The cause of exit for each doe was classified as a health-related death (endoparasitism, pneumonia, dystocia, etc.), culled and sold (infertility, injury, illness, etc.) or an accidental death (entrapment, predation, etc.) based on field notes. All deaths not attributed to accident were deemed illness-based. The product-limit method using the LIFETEST model of SAS (SAS Inst. Inc., Cary, NC) was used to test the significance of breed against survival trends. The Wilcoxon rank test was used to evaluate differences in population means. Frequencies of doe survival to each stayability endpoint and causes of doe exits among breed comparisons were further assessed by Chi-square analysis.

Lifetime production rates for each doe were evaluated at 2-, 3-, and 5-year stayability endpoints for total number of kids weaned and cumulative unadjusted kid weight weaned. Production was recorded as 0 for a doe in each year after exiting the herd. Analysis of survival and cumulative production was performed on the whole population. Total kid count weaned was also assessed under the alternative culling protocol. The subpopulation of does produced within the research herd was analyzed at 2- and 3-year stayability endpoints. Total kid count weaned was handled as a count response variable and analyzed using generalized linear mixed models for discrete data in the GLIMMIX procedure of SAS using the Newton–Raphson optimization technique with ridging. Number of kids weaned was tested using a Poisson distribution and log link function with means reported as generated by inversed link transformation to scale. Cumulative weight weaned and the difference in kid count weaned between the actual and alternative culling methods were analyzed using the MIXED model procedure in SAS. Statistical models included breed of dam as a fixed effect with year of entry and doe nested within doe breed as the error term to test doe breed. Least square means were compared using the Tukey-Kramer method (α = 0.05).

## Results and discussion

### Stayability

There were 201 doe exits which constituted 63.3% of the total herd inventory. Foundation does constituted 55.6% (n = 168) of the study population and 62.2% (n = 125) of total doe exits, thus older purchased does did not represent a disproportionate number of exits compared to younger replacement does produced within the herd. Health-related mortalities were the most prevalent cause of attritions (Table 2). With-in breed exit proportions were similar (P > 0.30) for each cause among breeds (Table 2). In sheep evaluations where culling for poor performance, conformation faults, and (or) age was practiced, half of ewe exits were attributed to health-related culls or deaths [17,21]. Norman and Hohenboken [22] reported 42% of ewe attritions over 4 years were illness-based with another 37% classified as unknown. Nugent and Jenkins [23] categorized half of ewe exits in an annual lambing system as physically disabled with 15 to 30% listed as diseased; however, physical conditions listed such as parasitism, foot rot, emaciation, missing, and unknown cause of death could easily have been considered as disease outcomes. A persistent condition of this herd that exhibited breed divergence was internal parasitism [18]. Goats are more susceptible to endoparasites than sheep under grazing conditions [24]. This should be considered when comparing doe and ewe attrition values.

**Table 2:** Reasons for doe exits from the study herd under the actual culling protocol

	**Breed**			
**Cause of exit**	**Boer**	**Kiko**	**Spanish**	**Cause total**
Death by illness, n^a^	89 (80.9)	39 (84.8)	37 (82.2)	165 (82.1)
Culled, n	16 (14.6)	5 (10.9)	4 (8.9)	25 (12.4)
Death by accident, n	5 (4.5)	2 (4.3)	4 (8.9)	11 (5.5)
Breed total, n	110	46	45	201

^a^Value in parenthesis is the proportion of the column exit total represented by a particular cause of exit. Breeds did not differ (*P* > 0.30) for proportion of exits within any cause.

Culls increased to half of all doe exits under the alternative culling protocol of doe removal at first reproductive failure (Table 3). Within-breed proportional exit values for each cause remained similar (P > 0.20) among breeds (Table 3). Removing does at first reproductive failure would reduce the proportional health-related exits by culling does earlier in life before an illness-induced death can occur. Culling at first failure would also increase the total exit number in the short term. First reproductive failure may also be a precursor to an impending health-related exit. A doe persistently thin may not wean a kid and would succumb later in the year or in the following year to a chronic condition like pneumonia or endoparasitism. The involuntary culling protocol of this project facilitated relative expression of fitness among the test breeds under the prevailing production conditions. Changes in culling strategy did not alter the profile of exit causes in Nugent and Jenkins [23] probably because of a no-cull policy for reproductive failure in the first production year for all culling plans and differences in classifying herd exits compared with the current study.

**Table 3:** Reasons for doe exits from the study herd under the alternative culling protocol

	**Breed**			
**Cause of exit**	**Boer**	**Kiko**	**Spanish**	**Cause total**
Death by illness, n^a^	57 (43.9)	32 (47.1)	29 (50.0)	118 (46.1)
Culled, n	71 (54.6)	34 (50.0)	26 (44.8)	131 (51.2)
Death by accident, n	2 (1.5)	2 (2.9)	3 (5.2)	7 (2.7)
Breed total, n	130	68	58	256

^a^Value in parenthesis is the proportion of the column exit total represented by a particular cause of exit. Breeds did not differ (*P* > 0.20) for proportion of exits within any cause.

Studies of longevity in small ruminants have generally included a minimum 6 years of observations. Past works vary in culling protocols that influence herd exits and in the chronological or physiological time unit used to define length of herd life. Studies on stayability in goats managed for meat production were not readily available in the scientific literature. Stayability rates across the breeds in the current study (61% at 3 years, 24% at 5 years) were similar to rates reported for sheep. Ewe stayability rates through 3 and 5 years were consistent across studies at 63 and 32% [21], 63 and 34% [1], and 62 and 22% [17]. In crossbred ewes, stayability rates were somewhat higher over 5 years at 68% with wide variation across 8 genotypes [25]. Stayability rates in dairy goats were generally lower. Tomar et al. [6] indicated stayability rates through 3 and 5 lactations were 27 and 5% respectively in Beetal does. Similarly, stayability through 3 years in Alpine does was 27% [26]. Lower values for dairy goats may be an indicator of high voluntary culling rates. However, Serradilla et al. [5] attributed low lactation numbers in dairy goats to very high disease-induced mortality rates. Dairy cattle breeds have lower longevity values than beef breeds [27].

**Figure 1:**
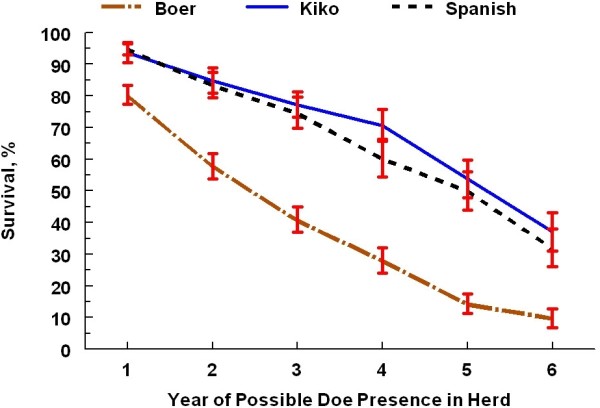
**Survival estimates (± se) for doe breeds using actual culling protocol of removal after second reproductive failure.** Boer differ (*P* < 0.001) from Kiko and Spanish for the survival curve over 6 years of production and for the stayability rate at each year of possible presence in the herd. Does in Year 1 were approximately 2–3 years of age. Does reaching Year 6 would have been approximately 7–8 years of age.

**Figure 2:**
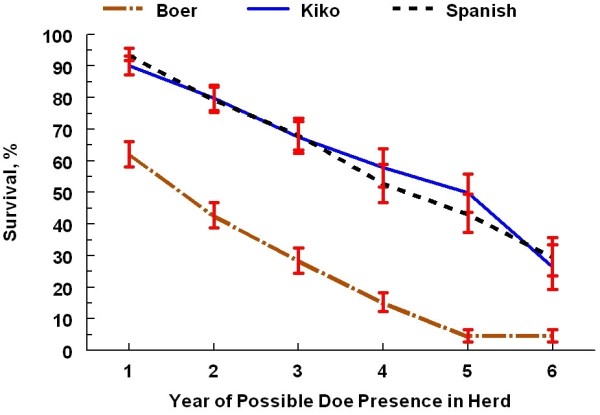
**Survival estimates (± se) for doe breeds using alternative culling protocol of removal after first reproductive failure.** Boer differ (*P* < 0.001) from Kiko and Spanish for the survival curve over 6 years of production and for the stayability rate at each year of possible presence in the herd. Does in Year 1 were approximately 2–3 years of age. Does reaching Year 6 would have been approximately 7–8 years of age.

The cumulative doe exit rate across the 6 calendar years of the project was higher (*P* < 0.01) for Boer does (83.3%) than for Kiko (50%) and Spanish (43%) does. The relationship among the doe breeds corresponds with the annual attrition rates reported earlier [18]. Boer does constituted the highest (*P* < 0.01) proportion (54.7%) of all exits compared with Kiko (22.9%) and Spanish (22.4%) does and the highest proportions of health-related mortalities and does culled and sold (Table 2). Boer does had a lower survival trend (*P* < 0.001) than Kiko and Spanish does and lower stayability rates (*P* < 0.001) at each endpoint (Figure 1); Kiko and Spanish did not differ for survival trend or stayability at any herd life endpoint. Stayability rates at 2, 3, and 5 years were of particular interest. All study does had the opportunity to complete at least 2 years of production. At 3 years, each doe had the opportunity to complete one round of the 3-breed mating diallel. Only foundation does made up the population subset with the opportunity to reach 5 years of herd life. Under the alternative culling protocol, Boer does still had the highest proportion (*P* < 0.01) of all exits (50.8%) compared with Kiko (26.6%) and Spanish (22.7%) does and the highest proportions of health-related mortalities and culled does (Table 3). Likewise, survival curves for the alternative culling protocol (Figure 2) showed a significant breed effect with Boer does having lower survival trends (*P* < 0.001) compared with Kiko and Spanish does and reduced stayability rates (*P* < 0.001) at each year of assessment. Kiko and Spanish does did not differ for survival trends under the alternative culling protocol (*P* = 0.94).

In agreement with this study, others have found breed differences for stayabilty rates among purebred females. Pèrez-Razo et al. [7] reported stayability rates from 4 to 6 years to be lower for Saanen does than for does of 4 other dairy goat breeds (Alpine, Granadina, Nubian, and Toggenburg). Cheviot ewes had lower stayability rates than 3 other sheep breeds from 5 to 8 years of production [28]. Columbia ewes had a higher stayability rate at 5 years compared with Targhee ewes [29]. Mutton Merino ewes had a higher stayability rate at 6 years compared with Dorper ewes [15]. A breed effect on longevity among purebred ewes was also found by Kern et al. [30], but not by Nawaz et al. [16]. Breed differences for stayability in small ruminants under conditions of minimal to no voluntary culling are linked to relative adaptedness to the production environment and associated disease resistance. Boer does exhibited higher annual rates of internal parasitism, lameness, reproductive failure, and attrition [18], all indicators of relatively poor fitness. The increased negative impact of reproductive failure shown under the alternative culling plan exacerbated the already negative relative outcome of Boer does. The divergence between Boer does and the other 2 breeds for longevity were evident from early in herd life and were persistent. Longevity is positively related to enterprise income in sheep [4,31]. The negative economic impact of early herd exits in ewes [32,33] is likely to be similar for meat goat does.

### Lifetime productivity

Total number of kids weaned and weight weaned per doe are shown for 3 stayability endpoints in Table 4. Consistent across year endpoints and parallel to doe stayability percentages, cumulative kid and weight production at weaning were significantly lower (*P* < 0.001) for Boer does compared with Kiko and Spanish does. Similar relationships favoring Kiko and Spanish were seen within the subpopulation of does produced within the research herd for cumulative production traits at 2- and 3-year stayability endpoints (data not shown). Total number of kids weaned remained lower for Boer does compared with Kiko and Spanish does under the more stringent alternative culling method (Table 5). The decline in cumulative kid productions from the actual to alternative culling protocol was similar among the three breeds. The difference between culling plans in kid count weaned for each doe breed differed significantly from 0 at 3- and 5-year stayability endpoints.

**Table 4:** **Effect of doe breed on whole-herd cumulative productivity at different stayability endpoints per available doe**^**a**^

	**Breed of doe**		
**Item**	**Boer**	**Kiko**	**Spanish**
	Stayability: Year 2		
Does available, n	132	92	79
Total kids weaned^b^, n	1.43 ± 0.18^x^	2.45 ± 0.31^y^	2.62 ± 0.34^y^
Total weight weaned^b^, kg	27.0 ± 1.8^x^	48.1 ± 2.2^y^	45.6 ± 2.4^y^
	Stayability: Year 3		
Does available, n	115	75	70
Total kids weaned^b^, n	2.08 ± 0.28^x^	3.60 ± 0.48^y^	3.84 ± 0.52^y^
Total weight weaned^b^, kg	39.3 ± 2.9^x^	70.7 ±3.5^y^	66.1 ± 3.7^y^
	Stayability: Year 5		
Does available, n	67	51	51
Total kids weaned^b^, n	3.24 ± 0.34^x^	6.09 ± 0.58^y^	6.21 ± 0.61^y^
Total weight weaned^b^, kg	57.1 ± 5.7^x^	110.8 ± 6.4^y^	99.8 ± 6.5^y^

^a^Does with opportunity to reach a stayability endpoint.

^b^Least square mean ± se.

^x,y^Means within a row not sharing a common superscript differ (*P* < 0.001).

**Table 5:** **Effect of doe breed on whole-herd cumulative kids weaned per available doe**^**a**^**under the alternative culling protocol**

	**Breed of doe**		
**Item**	**Boer**	**Kiko**	**Spanish**
	Stayability: Year 2		
Does available, n	132	92	79
Total kids weaned^b^, n	1.35 ± 0.18^x^	2.38 ± 0.32^y^	2.54 ± 0.37^y^
Change in total kids weaned^bc^, n	-0.05 ± 0.03	-0.05 ± 0.03	-0.05 ± 0.03
	Stayability: Year 3		
Does available, n	115	75	70
Total kids weaned^b^, n	1.95 ± 0.27^x^	3.37 ± 0.47^y^	3.64 ± 0.51^y^
Change in total kids weaned^bc^, n	-0.15 ± 0.06^*^	-0.24 ± 0.07^*^	-0.21 ± 0.08^*^
	Stayability: Year 5		
Does available, n	67	51	51
Total kids weaned^b^, n	2.87 ± 0.33^x^	5.42 ± 0.56^y^	5.91 ± 0.61^y^
Change in total kids weaned^bc^, n	-0.46 ± 0.15^*^	-0.71 ± 0.18^*^	-0.35 ± 0.18

^a^Does with opportunity to reach a stayability endpoint.

^b^Least square mean ± se.

^c^Change from the actual culling protocol.

^x,y^Means within a row not sharing a common superscript differ (*P* < 0.001).

^*^Mean within cell differs from 0 (*P* ≤ 0.01).

Previous studies on cumulative reproductive output among meat goat doe breeds were not found in the literature. Comparative studies have been reported for sheep. Nawaz et al. [16] reported greater cumulative lamb numbers and weights over 4 years for Polypay compared with Coopworth straightbred ewes. Mutton Merino ewes had higher 6-year cumulative weaned lamb number and weight output compared with Dorper ewes [15]. Columbia ewes had higher 5-year cumulative lamb number and weight weaned than Targhee ewes [29]. In agreement with purebred sheep results, differences were evident among straightbred meat goat does for cumulative reproductive output at weaning.

The cumulative reproductive output of the Boer, Kiko, and Spanish does paralleled the differences in longevity and was an extension of the previously reported comparison of whole-herd annual reproductive output [18]. The Kiko and Spanish does compared favorably with purebred ewe performance values for number of offspring weaned. Through 5 years of herd life, values reported for cumulative number of lambs weaned from straightbred bred ewes within breedtype comparison studies were 4.17 [17], 3.88 [34], and 3.77 lambs [35]. Conversely, the 5-year total number of kids weaned for Boer does was less than in the ewe studies. One caveat is that ewes were not allowed to raise more than 2 lambs in the older pair of studies if 3 or more lambs were born. However, it is unlikely that the opportunity to rear triplets would have elevated the reported 5-year lamb count weaned to levels similar to Kiko and Spanish does. In each of the sheep reports, purebred ewes weaned significantly fewer lambs over 5 years than F1 ewes. The 5-year total for kids weaned for straightbred Kiko and Spanish does also exceeded the F1 ewe totals of 5.66 [35], 4.85 [34] and 4.97 lambs [17]. Goats generally produce more offspring in a lifetime than sheep because of higher prolificacy rates [36]. Ewe studies on lifetime lamb production put into context cumulated kid production of the current goat study because comparable lifetime goat observations are not readily available in the scientific literature. Kiko and Spanish does supported Wilson [36] by producing more offspring than reported for ewes, whereas Boer does failed to do so.

### General

This report may be the first on breed differences among meat goat does for longevity and lifetime performance. There has been ample research done on longevity and lifetime performance comparing ewes breeds for lamb production. The body of sheep research served as a good reference to assess the results of this goat study with comparable outcomes realized. Reproductive output is considered a primary driver of profitability in meat animal production systems with herd health a major modulator of reproductive success. Cumulative kid weight weaned is the product of several components including the survival, fertility, and prolificacy of does and the survival and growth rates of kids through weaning. Except for the lack of a breed difference in doe prolificacy, Boer does were generally inferior for each contributing trait as presented in this and previous reports [18,20]. The relatively short herd life of Boer does is likely not conducive to recovering the costs associated with replacement doeling development and entry into the herd.

The 3 breeds were similarly responsive to the change in culling protocol. Núñez -Dominguez et al. [2] studied similar culling strategies in beef cows. Under most market conditions for calf and cull cow prices, a moderate plan similar to the actual culling protocol here was economically favorable compared with a more aggressive culling plan akin to the alternative protocol of this study under most market conditions. The negative economic impact of decreasing exit age from the optimal of 9 years in beef cows was also demonstrated [2]. In agreement, Nugent and Jenkins [23] concluded that intensive culling of ewes for reproductive failure, particularly before 3 years of age, was not beneficial to ewe flock productivity. Using approaches independent of but similar to Núñez -Dominguez et al. [2], exit ages between 4 and 5 years were considered optimal for bioeconomic efficiency in simulation models based on typical market conditions for ewes [37] and dual-purpose does [38]. Significant breed variation in cumulative economic return for ewes over 4 to 5 years with lifetime net revenue losses and gains was demonstrated among 8 crossbred genotypes with some environmental influences [25]. Economic models are sensitive to function assumptions and changes in input factors for animal and market components when generating outcomes. Nevertheless, it seems that the lifetime profiles of Kiko and Spanish does would produce more favorable results in bioeconomic efficiency models compared with Boer does.

The reason(s) for the relatively poor longevity and lifetime performance of the Boer does require further investigation. They were more prone to internal parasitism, lameness, and reproductive failure than Kiko and Spanish does [18]. Survivability was the basis of Spanish goat formation as a product of natural selection under dry climate conditions [13]. The Kiko breed was developed through artificial selection in a limited-input, wet climate environment with survivability a primary selection trait [14]. Kiko are somewhat similar to Spanish in that feral goats were used as the foundation of the Kiko composite breed. New Zealand feral goats are the product of natural selection. Selection in Boer goats has focused on enhanced conformation and increased size with seemingly lesser emphasis placed on fitness-related reproduction and survival traits [39,40]. Management inputs tend to be higher in Boer goat seedstock programs, although Boer goat germplasm generally flow into lower-input commercial doe herds. Higher maintenance requirements, lower immunological competencies, unfavorable feeding behaviors, and perhaps divergence between the doe breeds for major genes that affect fitness are possible contributors to the poor longevity and lifetime reproductive output of Boer does compared with Kiko and Spanish does observed here.

The relevance of reduced doe fitness becomes very apparent if lifetime reproductive output is considered as a trait of high economic significance in a meat goat enterprise [36,40]. Rauw et al. [41] and Van der Waaij [42] explained the negative fitness consequences of selecting for increased performance under optimal environmental conditions. Environmental sensitivities are heightened and rates of reproductive failure and illness increase when placing ‘improved’ germplasm in a suboptimal production environment. This scenario seems to fit the comparatively poor Boer results at this study location. Findings here are in opposition to earlier characterizations of the Boer goat as a hardy, disease-resistant breed that is adapted to a wide range of environmental conditions and unsurpassed for reproductive output [8,43]. The relatively greater propensity for illness in Boer goats leading to reduced lifetime productivity is exacerbated by the limited variety of pharmaceuticals approved for use in goat herd health management programs.

## Conclusions

The semi-intensive management plan used here was designed to assess doe performance under less than optimal pasture conditions typical of commercial meat goat programs in the region. The results of this study indicate stark differences among the meat goat breeds for long-term female fitness under the challenging environmental conditions typical of this geographical area. Sustained reproductive performance of Kiko and Spanish doe populations through 5 years of herd life (approximately 7 to 8 years of age) suggest that they would be preferable maternal breeds under production systems similar to the current conditions. Additional comparative research of Boer, Kiko, and Spanish does for lifetime traits under different environmental conditions will help to develop a more complete picture of their relative utility in meat goat production systems. Despite current use of Boer, Kiko, and Spanish goats in crossbreeding program, the comparable value of these 3 breeds as contributors to crossbred doe performance and general fitness remains to be determined under controlled conditions.

## Author contributions

RB was lead investigator in project design, herd management, and data collection over the six-year evaluation study. ANP took the lead in processing, analyzing, and interpreting the completed dataset. ANP and RB each made substantial contributions to preparing the final manuscript. All authors read and approved the final manuscript.
